# ROS Signaling and NADPH Oxidase in Red Algae

**DOI:** 10.3390/antiox14040480

**Published:** 2025-04-16

**Authors:** Gwang-Hoon Kim, Eunyoung Shim, Giuseppe C. Zuccarello

**Affiliations:** 1 Department of Biological Sciences, Kongju National University, Gongju 32588, Republic of Korea; tlasud@kongju.ac.kr; 2 School of Biological Sciences, Victoria University of Wellington, Wellington 6140, New Zealand; joe.zuccarello@vuw.ac.nz

**Keywords:** reactive oxygen species (ROS), NADPH oxidase, red algae, molecular phylogeny, calcium signaling

## Abstract

We explore the diverse roles of reactive oxygen species (ROS) and NADPH oxidases in red algae, focusing on their involvement in fertilization, wound repair, stress adaptation, and development. Once considered mere metabolic byproducts, ROS are now recognized as pivotal signaling molecules in red algae. ROS are actively produced and regulated by NADPH oxidase homologs in red algae. During fertilization, ROS facilitate gamete recognition and post-fertilization cell wall development. NADPH oxidase-produced ROS also play essential roles in wound repair, aiding cellular migration and cytoskeletal reorganization at injury sites. A survey of NADPH oxidase homologs in transcriptomic and genomic datasets shows that NADPH oxidase homologs have a long evolutionary history in red algae, with some orthologs duplicating before the split of the Bangiophyceae–Flordeophyceae, approximately 1.1 billion years ago. Additionally, we discuss modifications in red algal NADPH oxidase genes related to calcium binding compared to plants and hypothesize that independent calcium signaling pathways are involved. These insights reveal the significance of NADPH oxidase at a molecular level in supporting red algae’s physiological processes and adaptive strategies.

## 1. Introduction

ROS are chemically reactive molecules derived from molecular oxygen, produced as byproducts of cellular metabolism. While traditionally considered damaging byproducts, ROS have gained recognition as signaling molecules involved in various physiological and developmental processes across a broad range of organisms, including bacteria, plants, and animals [1,2,3]. Their dual nature, acting as both critical signaling agents and potential stressors, adds complexity to their role in cellular biology, requiring precise regulation to prevent oxidative damage. In eukaryotic cells, ROS are generated in organelles such as chloroplasts, mitochondria, and peroxisomes, with the NADPH oxidase (NOX or RBOH in plants) family mainly located on the cell membrane playing a significant role in their controlled production [4].

In terrestrial plants, ROS generated by NADPH oxidase play a crucial role in signaling pathways that regulate responses to both biotic and abiotic stresses, including pathogen attacks, drought, and salinity [5,6]. These ROS interact with other signaling molecules, such as calcium ions (Ca^2+^), to mediate defense responses, H_2_O_2_, to modulate growth, and regulate various developmental processes [7]. The NADPH oxidase complex, embedded in the plasma membrane, transfers electrons from NADPH to molecular oxygen, producing superoxide (O_2_^−^), which is subsequently converted into H_2_O_2_ either spontaneously or through enzymatic activity [8]. Hydrogen peroxide, among ROS, stands out for its stability and ability to traverse cellular compartments, making it an effective signaling molecule [9,10]. H_2_O_2_ also acts as a secondary messenger, modulating cellular responses through transcriptional regulation, protein phosphorylation, and calcium channel modulation [11,12].

In marine algae, ROS signaling extends beyond being mere metabolic byproducts to serve as regulators of stress responses and fundamental biological processes. Marine algae have evolved sophisticated systems for ROS production, scavenging, and signaling to adapt to dynamic and often harsh marine environments [13,14]. For instance, *Pyropia columbina* (formerly *Porphyra columbina*) demonstrates a complex antioxidant system that responds to desiccation through coordinated ROS signaling, which includes modulation of antioxidant enzymes and changes in photosynthetic performance [13].

The red alga *Gracilaria corticata* illustrates how marine algae use ROS signaling to adapt to varying salinity levels. Under different salinity conditions, *G. corticata* adjusts the activity of enzymes such as superoxide dismutase (SOD), catalase (CAT), and ascorbate peroxidase (APX), reflecting the complex nature of ROS-mediated stress responses [15]. This antioxidant network helps maintain redox homeostasis, enabling the algae to respond effectively to stress and enhance their survival in fluctuating marine environments. ROS signaling in algae also intersects with other pathways, such as calcium and hormonal responses. In the brown alga *Laminaria digitata*, oligoguluronate-induced responses trigger ROS-dependent signaling cascades, leading to the activation of specific gene expression patterns associated with defense mechanisms [16]. These responses showcase both conserved elements found in plant stress signaling and unique adaptations specific to marine settings.

Moreover, ROS play a crucial role in pathogen recognition and immune response activation, with NADPH oxidase-derived ROS amplifying signals and activating defense genes [17]. Environmental factors, such as seasonal variations, influence ROS signaling patterns. For example, studies on *Porphyra umbilicalis* (Rhodophyta) reveal that seasonal changes can affect antioxidant content and photosynthetic efficiency, highlighting the dynamic role of ROS in environmental adaptation [18]. These seasonal adaptations involve coordinated changes in photosynthetic components and antioxidant systems to cope with shifts in light and temperature.

Recent studies emphasize the potential of H_2_O_2_ as an indicator of seaweed stress, suggesting its application for monitoring seaweed health, particularly in aquaculture [19]. Beyond stress adaptation, ROS are implicated in regulating lipid metabolism and structural changes in thylakoid membranes [20]. Understanding the multifaceted roles of ROS in algae not only illuminates their adaptation strategies but also offers insights into their potential biotechnological applications.

## 2. Mechanisms of ROS Production and NADPH Oxidase Regulation

NADPH oxidases, or respiratory burst oxidase homologs in plants and algae, are membrane-bound enzymes that play a crucial role in generating ROS, specifically H_2_O_2_, as part of various cellular responses. Functioning at the plasma membrane, NADPH oxidase transfers electrons from cytosolic NADPH to molecular oxygen, producing superoxide anions (O_2_^−^), which are subsequently converted to H_2_O_2_ either spontaneously or through the action of superoxide dismutase (SOD) [4]. This ROS generation process is required for signaling pathways that regulate growth, development, and wound repair [6]. NADPH oxidase-derived ROS thus act as secondary messengers, amplifying cellular responses to environmental and physiological signals through modulation of ion channels, phosphorylation cascades, and transcriptional networks [2,6].

Red algae, like terrestrial plants, exhibit structural adaptations in their NADPH oxidase enzymes, which contribute to their functionality under specific environmental conditions. For instance, while RBOHs in land plants contain calcium-binding EF-hand domains that directly interact with Ca^2+^ to enable rapid activation, red algal NADPH oxidases often lack these domains [21]. Nevertheless, red algae can regulate ROS production through alternative mechanisms, possibly involving other cellular signals or ROS feedback loops [22]. This adaptability demonstrates the NADPH oxidase systems in red algae, allow them to respond effectively to various environmental stimuli despite structural differences with terrestrial plant counterparts.

Recent findings indicate that histone acetylation plays a significant role in modulating wound-induced responses and ROS signaling in red algae. A study demonstrated that inhibiting histone deacetylase (HDAC) activity in *Pyropia yezoensis* with suberoylanilide hydroxamic acid (SAHA) reduced the transcriptional activity of ROS-producing RBOH genes and attenuated ROS signals. This repression led to impaired spore formation and cell reprogramming post-wounding, highlighting the interplay between histone modifications and ROS-related cellular processes [23]. Such results underscore the regulatory complexity that exists between epigenetic mechanisms and ROS production pathways, broadening the understanding of how red algae adapt to environmental stresses.

## 3. Calcium and ROS Signaling in Algae

Calcium ions (Ca^2+^) serve as critical signaling molecules in both terrestrial and marine photosynthetic organisms, including algae, where they play a pivotal role in regulating ROS production [24]. In red algae, Ca^2+^-mediated signaling is essential for activating NADPH oxidase, which in turn influences ROS levels and various cellular processes. For instance, in *Griffithsia monilis*, a filamentous red alga, Ca^2+^-mediated ROS signaling has been observed to be crucial in cellular repair following mechanical damage. When cells of *G. monilis* are injured, NADPH oxidase activity increases, resulting in localized H_2_O_2_ production at the wound site. This ROS production serves as a signal for cell membrane resealing and cytoskeletal reorganization, both of which are necessary for effective repair [22]. Experimental studies using calcium channel blockers or ROS scavengers provide evidence that Ca^2+^ and ROS operate in a positive feedback loop. Ca^2+^ influx activates NADPH oxidase, generating ROS, which may subsequently enhance further Ca^2+^ channel activity [25]. This interplay between Ca^2+^ and ROS contributes significantly to the wound-healing responses observed in red algae, helping maintain structural integrity and cellular continuity within multicellular systems.

Recent findings by Shim et al. [24] reveal the intricate relationship between ROS and Ca^2+^ signaling during fertilization in *Bostrychia moritziana*. Their research demonstrates that Ca^2+^ influx activates calcium-dependent protein kinases (CDPKs), which subsequently regulate the ROS production necessary for successful fertilization. When either ROS production or Ca^2+^ influx was inhibited, the fertilization process was disrupted, highlighting this pathway’s critical role. This evidence points to a regulatory feedback mechanism, where Ca^2+^ signaling and NADPH oxidase activity are interconnected in red algae, despite these organisms lacking the direct calcium-binding domains typically found in plant RBOHs. Instead, red algae appear to utilize indirect calcium-sensing mechanisms, such as CDPKs, to modulate NADPH oxidase function under various physiological conditions [24].

## 4. ROS Signaling in Red Algal Reproduction and Development

In red algae, ROS play crucial roles throughout reproductive processes, acting as key signaling molecules that facilitate gamete recognition, attachment, and subsequent cellular events required for successful fertilization and development. In *Bostrychia moritziana*, ROS signaling is crucial for sperm–egg recognition, specifically through hydrogen peroxide production by NADPH oxidase in sperm cells. When spermatia (male gametes) come into contact with the trichogyne (the receptive part of the female reproductive structure), H_2_O_2_ is generated and diffuses into the egg cells, activating ROS-related signaling pathways mediating gamete fusion and initiating the genetic changes necessary for fertilization [26].

Beyond fertilization, ROS continue to influence post-fertilization development in red algae, facilitating cell wall formation, differentiation, and early zygote development. In *B. moritziana*, elevated ROS levels are maintained in cells surrounding the fertilized egg, aiding in zygote nourishment and initial developmental stages. Sustained H_2_O_2_ production by NADPH oxidase creates a localized redox environment that modifies cell walls, enhancing cellular differentiation and structural integrity [25]. This post-fertilization role of ROS parallels mechanisms in higher plants, where ROS produced during early development regulate transcription factors related to cell wall biosynthesis and embryo patterning, underscoring a conserved function in multicellular development across plant lineages [24].

## 5. Wound Response and Cell Repair Mechanisms in Red Algae

In red algae, NADPH oxidase-mediated ROS production is a critical component of the cellular response to physical injury, as exemplified in *Griffithsia monilis*, a species frequently studied due to its large easily wounded cells [22]. When cells within the filamentous structure of *G. monilis* are damaged, NADPH oxidase activity is rapidly upregulated at the wound site, resulting in a localized burst of hydrogen peroxide. This ROS burst acts as a signaling molecule to initiate repair responses, including elongation of neighboring cells and eventual somatic fusion, thereby closing the wound and restoring tissue continuity. This ability to respond swiftly to mechanical damage is vital for resilience in marine environments where physical stresses are common [25].

In red algae, wound healing refers to the coordinated cellular response that restores tissue integrity after injury, while cell repair involves the intrinsic recovery mechanisms within individual cells to maintain their structural and functional stability [22]. Cell repair in *G. monilis* also involves interactions between ROS and the cytoskeletal network, where ROS signaling facilitates the reorganization of microtubules and actin filaments, directing cell components toward the wound site. H_2_O_2_ not only stimulates repair cell elongation but also guides directional growth, allowing for effective migration and adhesion of repair cells. By coordinating ROS signals with cytoskeletal responses, *G. monilis* achieves efficient cell repair, which underscores the adaptive advantages of ROS in maintaining cellular integrity and structural resilience in red algae [22].

In addition, findings from *Pyropia yezoensis* highlight the broader regulatory role of ROS in cellular reprogramming and asexual reproduction following mechanical damage. Research shows that mechanical wounding induces a rapid increase in ROS production, mediated by NADPH oxidase, which facilitates wound-induced spore (WIS) formation. The use of an NADPH oxidase inhibitor, diphenyleneiodonium chloride (DPI), significantly suppresses ROS levels, subsequently inhibiting spore formation and the expression of genes related to cell cycle progression and dedifferentiation. This indicates that ROS signaling not only promotes immediate cell repair but also plays a critical role in reprogramming cellular function to adapt to environmental challenges, thus enhancing tissue resilience [27].

## 6. Molecular Phylogeny of Red Algal NADPH Oxidase

**Figure 1:**
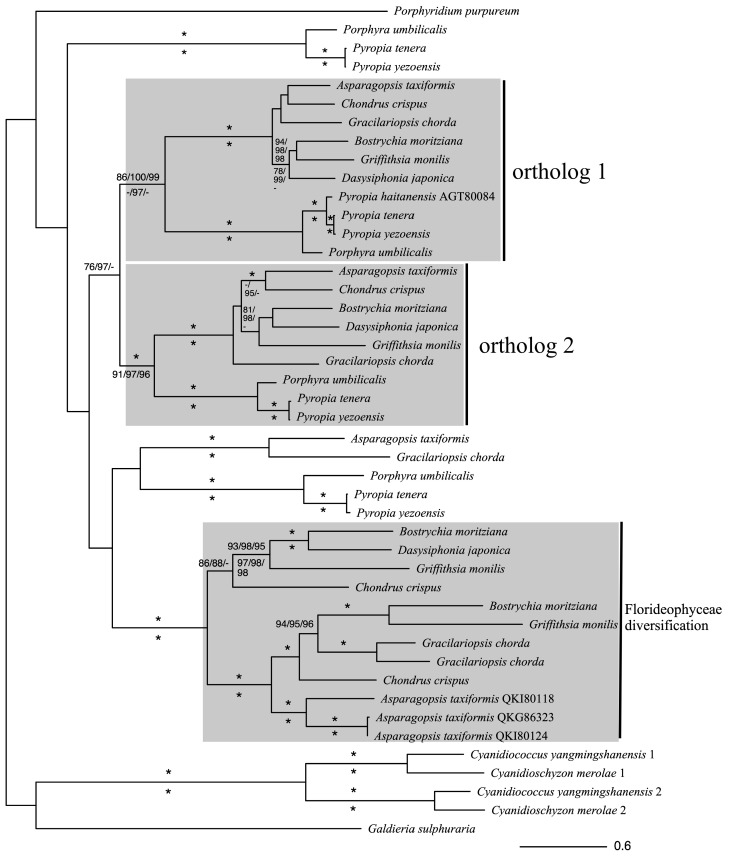
Maximum likelihood phylogeny of NADPH oxidase homologs in red algae. Alignment of amino acid sequences used MAFFT (1662 aligned positions), ML tree produced in IQTREE2. Support values for clades used non-parametric bootstrapping (500 replicates) [30]), ultrafast bootstrapping (UF) (2000 replicates), and the approximate likelihood ratio test (aLRT) (2000 replicates) above branches. A reduced alignment (TrimAL, 711 aligned positions) with ML support values below branch. Asterisks (*) indicate statistically significant bootstrap support values (≥95%).

The phylogeny of NADPH oxidase (Figure 1) in red algae shows the high diversity of these genes and the maintenance of gene duplications and expansion of this gene family, underlying the continued importance of these proteins in many aspects of red algal cell biology. Two supported sets of orthologous genes are resolved (orthologs 1 and 2), with both datasets, that reflect the known relationship between the two mayor groups of multicellular red alga (the Florideophyceae, e.g., Griffithsia, and the Bangiophyceae, e.g., Pyropia) and genera within these classes (for example, grouping *Bostrychia*, *Griffithsia*, and *Dasysiphonia* from the order Ceramiales). This ortholog duplication must have occurred in the common ancestor of these two classes. As the Bangiophyceae are one of the earliest known identified fossil algae, this duplication occurred before 1 billion years ago [28,29].

There is also a highly diverse supported clade containing sequences from Florideophyceae red alga. The Florideophyceae are considered ‘complex’ red algae, as they show a high level of morphological complexity, a complex reproductive life cycle and zygote development, and are the most abundance and speciose of all the red algal groups [31]. The relationships within this group show two clades corresponding to the known phylogenetic relationships of the red algae, suggesting that a further duplication occurred before the diversification the Florideophyceae. Several other copies of NADPH oxidase are seen in red algae that do not form any particular clades.

The high diversity of NADPH oxidase is particularly evident in red algae when comparing genomic data to transcriptomic data. The transcriptome of *Pyropia tenera* (Bangiophyceae) has four orthologs in our dataset while the genome of *Asparagopsis taxifomis* has six. Further genomes and integration of gene duplications may more fully determine the evolution of the NADPH oxidase gene family in the red algae.

## 7. Comparative Analysis of RBOH Genes

**Figure 2:**
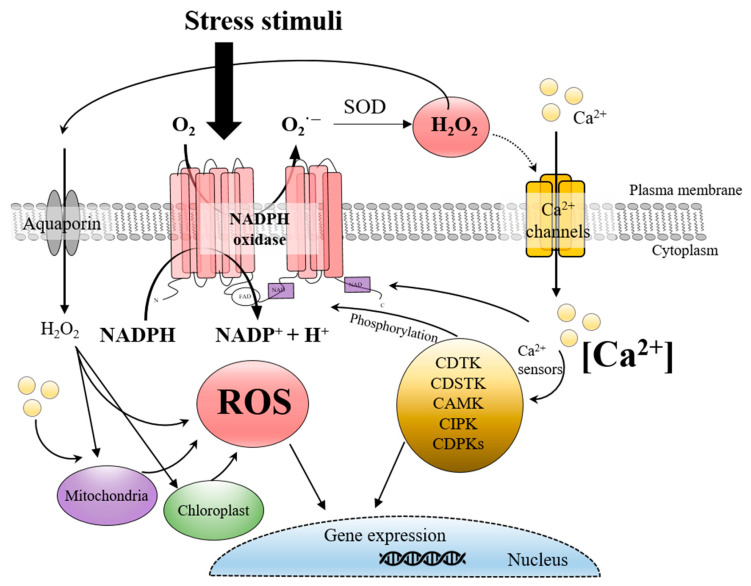
Schematic diagram of the elements involved in the redox signaling mediated by NADPH oxidase found in red algae to date. NADPH oxidase on the plasma membrane converts molecular oxygen (O_2_) to superoxide anions (O_2_^−^), which transform into hydrogen peroxide (H_2_O_2_) via superoxide dismutase. H_2_O_2_ functions as a membrane-permeable secondary messenger across cells. Unlike terrestrial plants with calcium-binding EF-hand domains, red algal NADPH oxidases are regulated by alternative calcium-dependent mechanisms, likely through calcium-dependent protein kinases (CDPKs). This creates a feedback loop where calcium activates NADPH oxidase, producing ROS that may enhance calcium signaling.

The evolution of respiratory burst oxidase homologs, including NADPH oxidase, highlights adaptations that allow diverse organisms to harness ROS for varied biological functions. In plants and algae, RBOHs have diversified to support cellular processes for survival in different environments (Figure 2). While terrestrial plants possess RBOHs with calcium-binding EF-hand motifs that directly interact with calcium ions (Ca^2+^), facilitating rapid responses to environmental stress, red algal RBOHs often lack these motifs. Despite this structural variation, red algae have evolved alternative regulatory mechanisms to control NADPH oxidase activity, enabling efficient ROS production and signaling within saline marine habitats [21].

In algae, particularly red algae, NADPH oxidase plays diverse functional roles, including managing salinity stress, facilitating wound repair, and supporting reproduction, highlighting its adaptations to the marine environment. Studies suggest that NADPH oxidase contributes to cellular homeostasis under high salinity by modulating ROS-mediated antioxidant responses and ion transport. Similar to halophytic plants that use NADPH oxidase to regulate osmotic balance and ROS signaling in saline conditions, red algae exhibit parallel adaptations that enhance their resilience in fluctuating salinities [32].

This evolutionary flexibility underscores the broad utility of NADPH oxidase across both aquatic and terrestrial lineages, illustrating how adaptations in ROS signaling pathways enable organisms to meet the demands of their respective ecosystems. Further understanding of these evolutionary adaptations provides valuable insights into how ROS signaling supports the survival and functional versatility of algae in diverse marine environments.

## 8. Functional Diversity of NADPH Oxidase

The functional scope of NADPH oxidase has expanded significantly across photosynthetic lineages, reflecting evolutionary adaptations to diverse ecological pressures. In land plants, NADPH oxidases are central to immune responses, generating ROS during pathogen attack to initiate the hypersensitive response, isolate infected cells, and trigger defense signaling in surrounding tissues [11]. This role is well established in terrestrial environments where rapid and localized defense is vital.

In contrast, the functions of NADPH oxidase in algae, particularly red algae, reflect adaptations to unique marine challenges. While pathogen defense has not been extensively studied in red algae, recent evidence suggests that NADPH oxidase may participate in defense responses to oomycete pathogens through calcium-mediated ROS signaling [33,34]. These findings indicate that immune-related functions of NADPH oxidase may also be conserved, although differentially regulated in marine systems.

Beyond immunity, NADPH oxidase participates in a range of developmentally regulated and stress-responsive processes across plant and algal taxa. In terrestrial plants, it is involved in regulating root hair development, stomatal closure, and overall growth dynamics, linking ROS signaling to morphogenesis and physiological plasticity [7]. In green algae such as *Dunaliella salina*, NADPH oxidase-driven ROS signaling supports photoprotection by mediating carotenoid biosynthesis under high light stress [35].

In red algae, NADPH oxidase has been shown to regulate species-specific physiological processes such as fertilization (*Bostrychia moritziana*) and wound-induced cell fusion and regeneration (*Griffithsia monilis*), demonstrating its role in ensuring reproductive success and structural integrity under environmental stress [25,26]. Notably, red algae lack some of the classical calcium-binding motifs found in plant RBOHs, yet maintain ROS production through alternative regulatory systems, underscoring the enzyme’s evolutionary flexibility.

## 9. Conclusions and Future Directions

Understanding the mechanisms of ROS production and NADPH oxidase function in red algae offers significant insights into how these organisms adapt to the dynamic and often challenging marine environment. NADPH oxidase-mediated ROS signaling supports critical biological processes, including stress adaptation, wound repair, and reproductive development, underscoring its versatile role in maintaining cellular resilience. These insights reveal broader implications for stress biology, particularly in the context of environmental shifts affecting marine ecosystems.

Future research should investigate the unique regulatory pathways governing NADPH oxidase activity in red algae, especially considering the absence of direct calcium-binding domains that are present in terrestrial plant counterparts. Investigating alternative calcium-dependent or indirect regulatory mechanisms could uncover novel ways that red algae control ROS production in response to environmental stress.

Comparative genomics offers a promising avenue for identifying NADPH oxidase homologs and unique regulatory genes across algal species, providing deeper evolutionary insights and potentially unveiling new genes relevant to stress tolerance, cellular repair, and development. Additionally, understanding the balance between ROS production and antioxidant activity in red algae could lead to applications in marine biotechnology, where optimized redox management may enhance the health and productivity of cultivated algae.

Overall, further studies into NADPH oxidase and ROS signaling pathways in red algae will not only deepen our knowledge of their adaptive strategies but also open pathways for practical applications in sustainable aquaculture and environmental resilience.

## Data Availability

The original contributions presented in this study are included in the article. Further inquiries can be directed to the corresponding author.

## Abbreviations

The following abbreviations are used in this manuscript:

ROS	Reactive Oxygen Species
NADPH Oxidase	Nicotinamide Adenine Dinucleotide Phosphate Oxidase
RBOH	Respiratory Burst Oxidase Homolog
H_2_O_2_	Hydrogen Peroxide
O_2_^−^	Superoxide Anion
SOD	Superoxide Dismutase
CAT	Catalase
APX	Ascorbate Peroxidase
CDPKs	Calcium-Dependent Protein Kinases
DPI	Diphenyleneiodonium Chloride
HDAC	Histone Deacetylase
WIS	Wound-Induced Spore
